# The Threat of Adversarial Attack on a COVID-19 CT Image-Based Deep Learning System

**DOI:** 10.3390/bioengineering10020194

**Published:** 2023-02-02

**Authors:** Yang Li, Shaoying Liu

**Affiliations:** Graduate School of Advanced Science and Engineering, Hiroshima University, Higashi-Hiroshima 739-8511, Japan

**Keywords:** adversarial attack, COVID-19, deep learning, security

## Abstract

The coronavirus disease 2019 (COVID-19) rapidly spread around the world, and resulted in a global pandemic. Applying artificial intelligence to COVID-19 research can produce very exciting results. However, most research has focused on applying AI techniques in the study of COVID-19, but has ignored the security and reliability of AI systems. In this paper, we explore adversarial attacks on a deep learning system based on COVID-19 CT images with the aim of helping to address this problem. Firstly, we built a deep learning system that could identify COVID-19 CT images and non-COVID-19 CT images with an average accuracy of 76.27%. Secondly, we attacked the pretrained model with an adversarial attack algorithm, i.e., FGSM, to cause the COVID-19 deep learning system to misclassify the CT images, and the classification accuracy of non-COVID-19 CT images dropped from 80% to 0%. Finally, in response to this attack, we proposed how a more secure and reliable deep learning model based on COVID-19 medical images could be built. This research is based on a COVID-19 CT image recognition system, which studies the security of a COVID-19 CT image-based deep learning system. We hope to draw more researchers’ attention to the security and reliability of medical deep learning systems.

## 1. Introduction

Towards the end of 2019, the coronavirus disease 2019 (COVID-19), caused by the SARS-CoV-2 virus, emerged; infections were mainly transmitted through respiratory droplets, spread rapidly, and eventually, were recognized as a global pandemic by the WHO [1,2]. The most common clinical symptoms of COVID-19 infection include a cough, fever, headache and so on [3,4]. Particularly, in high-risk populations such as the elderly or those with numerous disorders where COVID-19 may induce lung damage, infection with COVID-19 is more likely to result in viral pneumonia. Severely infected patients may develop acute respiratory distress syndrome, severe lung infection and fibrosis, and even death [5]. The rapid global spread of COVID-19 has caused serious damage to human health, the world economy, and public health security [6,7,8].

At the beginning of the COVID-19 epidemic, its clinical diagnosis was based on a patient’s epidemiology, clinical presentation, a chest X-ray, chest CT, and RT-PCR [9,10]. As compared with other diagnostic methods, chest CT, which is the main tool for screening and diagnosing COVID-19, can detect pulmonary lesions and can also classify patients into early, intermediate, or severe cases based on CT manifestations in the chest [11,12,13]. CT images of the lungs of patients with COVID-19 show patchy or ground glass shadows [14]. As the disease progresses, the severity of the lung lesions may become more significant, and pulmonary fibrosis may develop, with a white coloration of both lungs detected by CT lung examination [15,16]. Therefore, it is crucial to display information about the lungs of COVID-19 cases through CT. Doctors can correctly evaluate patients’ CT images, thus, diagnosing patients’ conditions for early detection and treatment.

COVID-19 has attracted considerable attention from researchers, and studies on the disease have covered various fields such as informatics, biochemistry, medicine, and vaccines [17,18,19,20]. One of the most exciting topics has concerned the application of machine learning in the study of COVID-19 [21,22]. Narayana Darapaneni proposed a machine learning approach to predict COVID-19 cases in a clinical test sample population with an accuracy of 87.0–97.4 percent [23]. Aras M. Ismael built a new deep learning model in order to detect chest X-ray images of COVID-19 [24]. Nathalie Lassau combined a CT image deep learning model with biological and clinical information to more accurately predict the extent of COVID-19 patients’ diseases [25]. Pahar M developed a machine learning model to discriminate between COVID-19 positive coughs and COVID-19 negative coughs captured on smartphones. The Resnet50 classifier performed the best out of seven machine learning classifiers that were separately examined and tested, and it could be a viable and practical technique for non-contact COVID-19 screening [26]. Wang, X. developed a weakly supervised deep learning model for the identification and lesion localization of COVID-19 cases using 3D CT volumes. The accuracy of the model was 0.901 for the classification of positive and negative COVID-19. It offered a quick and reliable approach for the quick identification of COVID-19 patients [27]. Shervin Minaee trained four well-known convolutional neural networks using transfer learning to analyze chest X-ray images and to detect COVID-19 infection. The majority of these networks attained remarkable performance [28]. Mohit Agarwal used nine artificial intelligent models, including machine learning, deep learning, and transfer learning, to classify and to characterize COVID-19 lung CT. As compared with other methods, deep learning showed superior performance in analyzing the severity of COVID-19 lung infections [29]. Wang, L. considered available chest X-rays of COVID-19 patients and suggested a COVID-Net model based on a deep convolutional neural network that could identify COVID-19 cases from chest X-rays. The model could aid doctors in improving screening to identify COVID-19 cases [30].

There is no doubt that deep learning has achieved remarkable success in many fields such as autonomous driving [31], e-commerce [32], medical image analysis [33], and protein structure prediction [34]. A medical image analysis is the study of interactions with the human body using a specific media. Then, the internal organs and tissues of the human body are represented as images, and diagnostic doctors make decisions based on the data supplied by medical images to evaluate the body’s health. Current common medical imaging includes X-ray imaging, computed tomography, nuclear magnetic resonance imaging, cardiac angiography, mammography, and so on. Medical image diagnosis has undergone a revolution thanks to deep learning. However, the security of deep learning systems has also received considerable attention from researchers [35]. Szegedy et al. [36] first identified the weaknesses of deep neural networks in image classification tasks, whereby, input samples that were intentionally added with some subtle human-imperceptible perturbations to the input samples were shown to give the model an incorrect output with high confidence. More unexpectedly, the same image perturbation was shown to fool many network classifiers, and this interesting phenomenon has encouraged many researchers to pay more attention to issues such as the security of deep learning [37,38]. However, most of the current research on the security of deep learning has focused on natural images and natural language, and there has been little research on medical images. As compared with other deep learning systems, the security of medical image-based deep learning systems is crucial, because medical images are related to personal health data, and the correct recognition and analysis of medical images through deep learning systems is an important tool to assist doctors in diagnosis, and any wrong diagnosis about a condition may bring irreparable harm. Additionally, dishonest individuals could attack the medical deep learning system in an effort to tamper with the outcomes of medical image diagnosis, which would subsequently lead to insurance fraud. Therefore, in this paper, we study the security of a COVID-19 CT image-based deep learning system.

The main contributions of this paper are as follows:

1. We built a deep learning system based on COVID-19 CT images and non-COVID-19 CT images, and the model achieved good performance for the classification of two different CT images with an average accuracy of 76.27%.
2. We used an adversarial attack algorithm, FGSM, to demonstrate the existence of security vulnerabilities in the COVID-19 CT image-based deep learning system. The pretrained model’s classification accuracy of non-COVID-19 CT images decreased from 80% to 0% when FGSM was used to attack it.
3. To address the security vulnerabilities of medical image-based deep learning systems, we discussed how to build a COVID-19 CT-based deep learning system with good defense performance.

## 2. Related Work

In this section, we introduce the concept of the adversarial sample, the classification of attack methods, and methods of generating adversarial samples.

### 2.1. Adversarial Sample

An adversarial sample is generated by applying subtle perturbations (that are difficult to detect by the naked eye but are acceptable to the deep learning model) to the original data, leading to the input data being misjudged by the deep learning model. The input data are denoted by *x*, the deep learning model is denoted by *g*, the classification result is denoted by *g*(*x*), and the perturbation is denoted by *ϵ*. Suppose there is a slight perturbation *ϵ*:

|*ϵ*| < *δ and* g(*x* + *ϵ*)! = *g*(*x*)

Then, *x* + *ϵ* can be called an adversarial sample.

### 2.2. Classification of Methods

There are various classifications of attacks based on their attack environments; therefore, attacks can be classified as black-box, white-box, and gray-box attacks [39].

Black-box attacks mean that the attacker does not know the internal structure of the attacking model, the training parameters, or the defense methods, and can only interact with the model through the output.

White-box attacks are unlike black-box models, as the attacker knows everything about the model, including the network structure and parameters. Most of the current attack algorithms are white-box attacks.

Gray-box attacks are found between black-box and white-box attacks, and only a part of the model is known (e.g., realizing the output probability of the model or understanding the model structure but not the parameters).

In relation to the purpose of the attack, attacks can be divided into targeted and untargeted attacks [40].

An untargeted attack is associated with image classification, namely in the sense that the attacker only needs to make the target model misclassify the sample but does not specify which classification is wrong.

A targeted attack means that the attacker specifies a class so that the target model not only misclassifies the samples but also misclassifies them into the specified type. In terms of difficulty, targeted attacks are more challenging to implement than untargeted attacks.

### 2.3. Current Methods of Generating Adversarial Samples

There are several adversarial attack methods proposed in the literature, but we only discuss the ones that are most relevant in this section.

#### 2.3.1. Optimization-Based Generation of Adversarial Samples

In the training phase of the model, the value of the loss function is continuously reduced by calculating the loss function between the predicted and true values of the sample data, adjusting various parameters of the model in the backward transfer process, and iteratively calculating the parameters of each layer of the model to generate adversarial samples. Carlinr et al. [41] proposed a set of adversarial C&W attacks based on optimization, considering both a high attack rejection rate and low adversarial disturbance.

$$r_{n}=(tanh(w_{n})+1)/2-x^{n}\;\min_{wx}\|r_{n}\|=c\;f\;((tanh(w_{n})+1)/2)\;where\;f(x^{’})=max(max\{Z(x^{’})i_{x}:i\neq t\}-Z(x^{’})t,-k)$$

#### 2.3.2. Gradient-Based Generation of Adversarial Samples

The gradient is obtained from the input data in the training phase, then the input data are updated stepwise according to the loss function, and finally, the adversarial sample is obtained. Commonly used adversarial attack algorithms include the fast gradient sign method (FGSM) [42], the basic iterative method (BIM) [43], and the project gradient descent (PDG) method [44]. The specific form of the adversarial samples generated with the FGSM is as follows:

*x∗* = *x* + *ϵ* · *sign*(∇*x J*(*θ*,*x*,*y*))

where *x* is the input data, *y* is the label of *x, θ* is the parameter of the model, *J*() is the loss function, and *ϵ* is an artificially set perturbation parameter. The FGSM algorithm is shown below (Algorithm 1).
**Algorithm 1** FGSM.Input: original image, orig_im; original_target, orig_tar; Output: adversarial image, adv_im; adversarial target, adv_tar; adv_im = orig_im iteration = 1 **while** iteration < max_iteration and adv_tar = orig_tar adv_im = orig_im + iteration * step_size * sign(gradient(orig_im)) adv_im = clip(adv_im, min, max)  iteration + = 1 **end** **return** adv_im

#### 2.3.3. Adversarial Network-Based Generation of Adversarial Samples

In 2014, Goodfellow proposed exciting adversarial attack networks (GANs) [45], and then various studies on GANs have also emerged. GANs consist of two parts: a generator and a discriminator. A generator (G) is used to generate realistic samples from random noise, and a discriminator (D) is trained to discriminate the real data from the generated data, and both are trained at the same time until a balance is reached, in which the data generated by the generator is indistinguishable from the real data, and the discriminator cannot distinguish the generated data from the real data correctly. Similarly, GAN-based networks can generate adversarial samples more efficiently. AdvGAN is a method for generating adversarial samples based on GANs models; given the input *x*, the perturbation *G*(*x*) is generated by the generator network [46]. On the one hand, *G*(*x*) *+ x* is sent to the discriminator network for training, and on the other hand, *G*(*x*) *+ x* is sent to the attacked network. The objective loss function is continuously optimized, and *G*(*x*) is the perturbation when the model reaches optimality. The target loss can be decomposed into three parts, expressed as:

$$L=L_{adv}^{f}+\alpha L_{adv}+\beta L_{hinge}$$

where $L_{adv}^{f}$ is the misleading misclassification loss, *L_adv_* is the loss function of the GAN, and *L_hinge_* is used to restrict the perturbations to a certain range.

We summarized the main adversarial attack algorithms in Table 1.

## 3. Experiment

The experiments in this paper consist of two parts, building a deep learning system and attacking the deep learning system. To demonstrate the security vulnerability and attack ability of a deep learning system based on COVID-19 CT images, first, we built a deep learning system that could accurately identify CT images infected with COVID-19 and CT images without COVID-19 infection, and then we attacked this deep learning system with the adversarial attack algorithm FGSM.

### 3.1. Building the Deep Learning System

**Figure 1:**
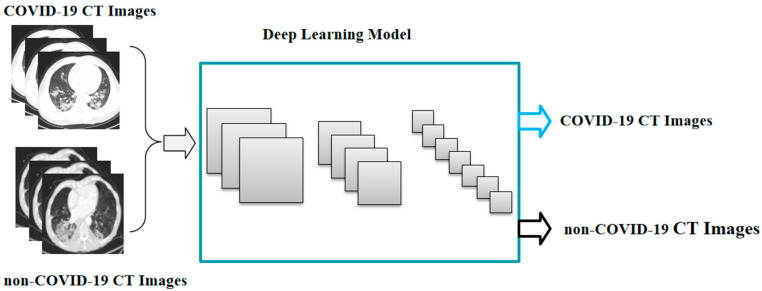
The pipeline of the COVID-19 CT image-based deep learning model.

Building the deep learning system involved training and testing a deep neural network (Figure 1). The training and testing stages meant that datasets had to be selected. The deep neural network needed to be carefully selected. In this section, we address these issues.

#### 3.1.1. Datasets

The CT image data in this paper were obtained from publicly available datasets extracted from the medRxiv and bioRxiv preprints of COVID-19 by Xingyi Yang at the University of California, San Diego [52]. These datasets are anonymous and can be applied to the study of COVID-19. The datasets contained 349 CT images of COVID-19 infection cases (COVID-19 CT images) and 397 CT images of cases without COVID-19 infection (non-COVID-19 CT images). The whole dataset was divided into three parts (the training set, the validation set, and the testing set), with a ratio of 0.8:0.1:0.1 (Table 2).

#### 3.1.2. Deep Learning Model

As compared with machine learning, the advantage of deep learning is that the network capacity is large enough to accommodate richer feature information, and the deep learning effect always improves as the number of data increases and deepens. Deep learning is a complex machine learning algorithm, and with continuous research, many classical deep learning models have emerged, which have greatly improved the performance of deep learning. We chose the classical Resnet model, the winning model of ImageNet 2015, which offers several advantages such as a very low error rate; it also presents little complexity and only requires small computational effort [53]. One of the factors for better performance of deep learning is the dataset; a large dataset can make the model achieve better training results. Transfer learning is a powerful method for transferring knowledge learned in one scenario to another scenario application. Since there are fewer CT images in the public dataset, it is difficult to achieve better performance of a deep learning model based on such a small dataset if trained from scratch; therefore, transfer learning can help to train a deep learning model with better performance more efficiently. Therefore, we used the transfer learning method to build a deep learning system based on COVID-19 images using the pretrained Resnet-50 model. The parameters of the model were frozen, the pooling layer and fully connected layer were replaced, and the dropout layer rate was set to 0.5. The optimizer used adaptive moment estimation (Adam) [54], performed fine tuning using stochastic gradient descent with a learning rate of 1 × 10^-3^, and fully changed the connected layer to two classifications (COVID-19 CT images and non-COVID-19 CT images). Preprocessing and data augmentation operations were performed on all CT image datasets.

#### 3.1.3. Metrics

The performance metrics used in this paper to evaluate the COVID-19 image-based deep learning model were accuracy and area under the curve (AUC) [55]. True positives (TP) indicated the number of COVID-19 images that were correctly classified as COVID-19 CT images. False positives (FP) indicated the number of non-COVID-19 images that were incorrectly classified as COVID-19 images. True negatives (TN) denoted the number of non-COVID-19 images that were correctly classified as non-COVID-19 CT images. False negatives (FN) indicated the number of COVID-19 images that were incorrectly classified as non-COVID-19 CT images.

Accuracy = (TP + TN)/(TP + TN + FP + FN)

### 3.2. Adversarial Attack of the COVID-19 CT Image-Based Deep Learning System

**Figure 2:**
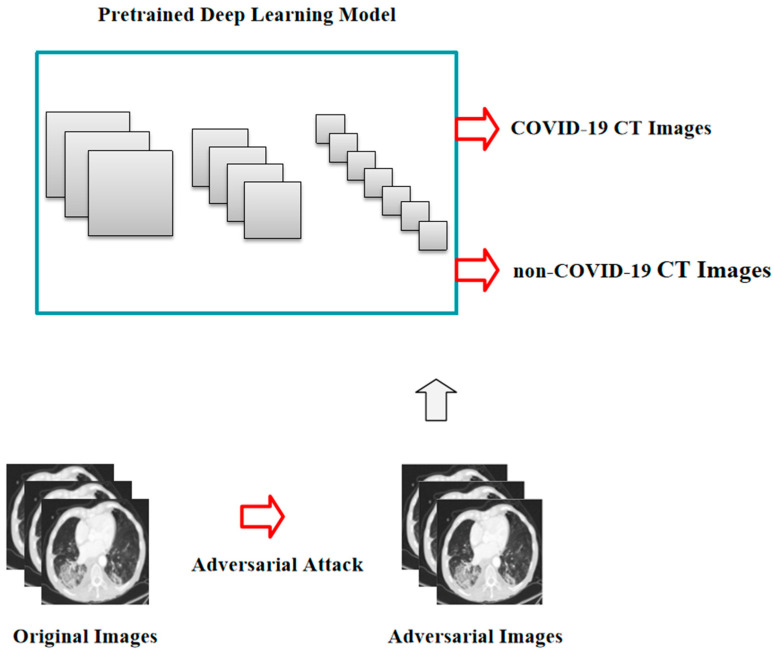
The pipeline of adversarial attack against the COVID-19 CT image-based deep learning system.

To verify the security and reliability of the deep learning model based on COVID-19 CT images, we attacked the pretrained model by adding subtle interferences to the non-COVID-19 CT images of the testing set, which were hard for the naked eye to detect and could be misclassified by the model (Figure 2).

In addition, in order to verify the relationship between the epsilon of the adversarial attack algorithm and the classification accuracy of the deep learning model, we took non-COVID-19 CT images as the study object and tested the effect of different epsilons on the classification accuracy of the model. Based on the pretrained deep learning model that could correctly classify COVID-19 CT images and non-COVID-19 CT images, we used the pretrained models with the FGSM algorithm based on a gradient to generate adversarial images.

## 4. Results

After training, we tested and obtained an accuracy of 76.27% and an AUC value of 85.80% for the COVID-19 CT image-based deep learning model (Table 3). This indicates that the model can accurately identify COVID-19 CT images and non-COVID-19 CT images and possesses good recognition accuracy.

**Figure 3:**
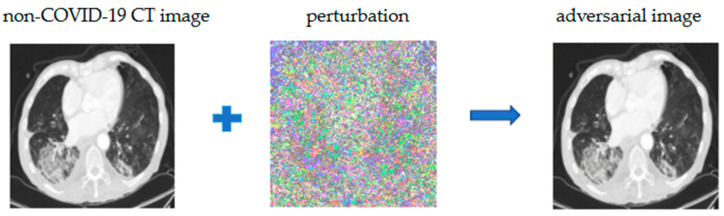
Characteristic results of adversarial image and non-COVID-19 image.

Based on the above-trained model, we took a non-COVID-19 infection as an example and superimposed a slight perturbation on the original image with the FGSM algorithm. The deep learning model does not correctly classify non-COVID-19 images, and it is also difficult to discriminate with the human eye when we compare the adversarial image with the non-COVID-19 image. (Figure 3).

**Figure 4:**
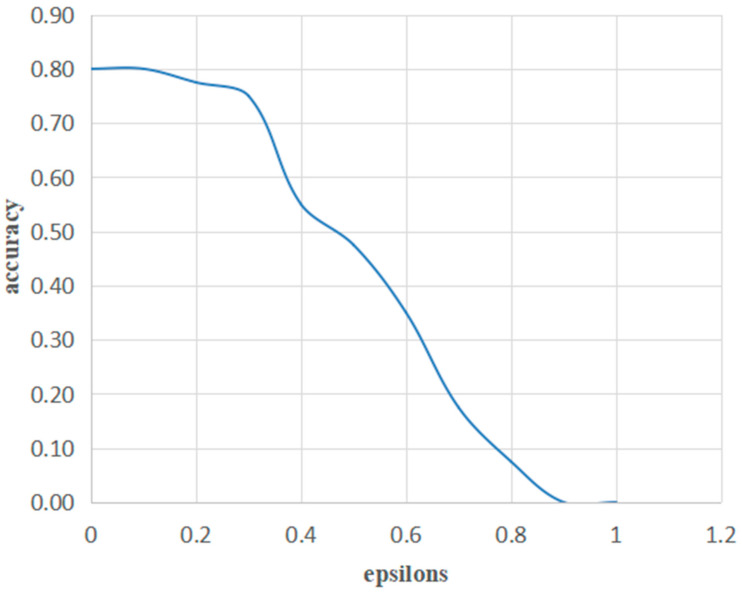
The accuracy for different epsilons.

To investigate the perturbation of epsilons on the accuracy of the testing set, we set different epsilon values, and then evaluated the relationship between the epsilons and the accuracy of the non-COVID-19 CT images (Table 4). As shown in Table 4, when epsilon is 0, the deep learning system is not attacked by the adversarial attack algorithm FGSM and the accuracy of the model is 80%, which indicates that the model has good recognition performance for non-COVID-19 CT images. We found that the accuracy of the test set decreased with increasing epsilons, suggesting that superimposing a larger epsilon on the original data could allow the deep learning model to classify images with a higher error rate (Figure 4).

**Figure 5:**
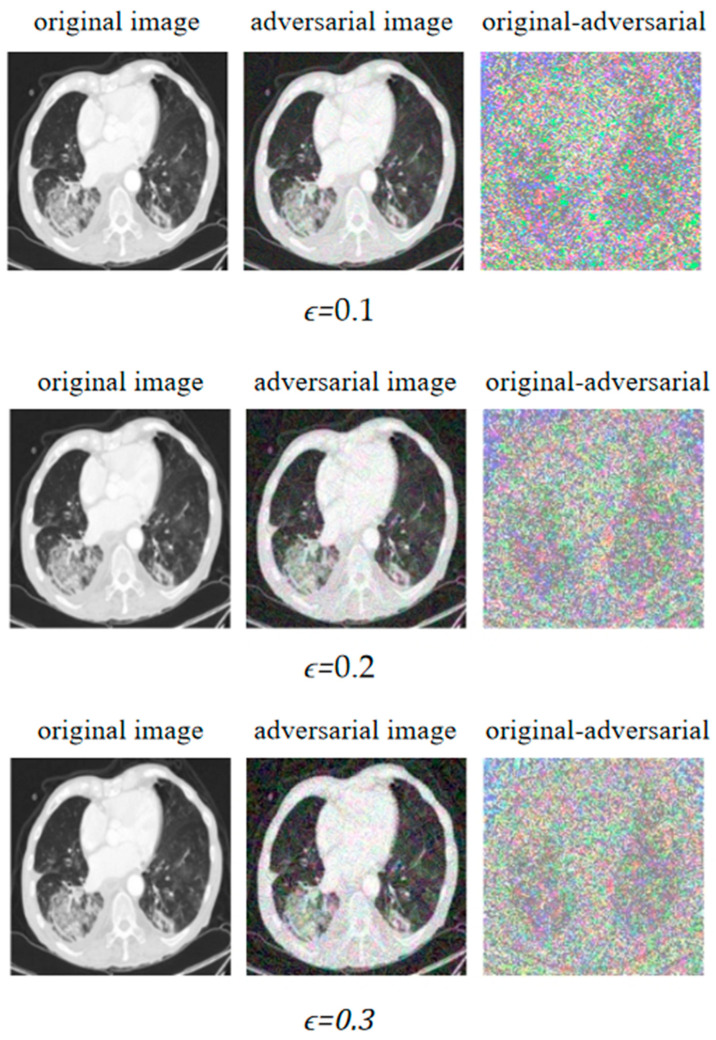
The FGSM attack on original images under different epsilons.

To illustrate that the adversarial attack could degrade the detection performance of the COVID-19 CT image-based deep learning system, we took the FGSM attack as an example and conducted experiments by setting different perturbation rates in the original image to verify the perturbation change and display the corresponding CT images. As shown in Figure 5, when the perturbation was set to 0.1, 0.2, and 0.3, all the adversarial images could be successfully generated. However, when the perturbation ϵ was 0.1, an adversarial image that was unrecognizable to the human eye could be successfully generated, while the generated adversarial image could be recognized by the human eye when the epsilon was set to 0.3. Therefore, in the study of adversarial attacks, the balance between the classification accuracy of the adversarial attack on the deep learning system and the recognition accuracy of the adversarial image by the human eye is a very important issue. We want the adversarial image to fool the deep learning model with a high fooling rate, but not be easily detected by the human eye.

## 5. Discussion

We analyzed and studied the impact of adversarial images on deep learning recognition based on COVID-19 CT images. While most previous studies on deep learning for COVID-19 have focused on how to build a deep learning system that was capable of accurately recognizing COVID-19 CT images, we focused on the security and reliability of the deep learning system based on COVID-19 CT images. By implementing the transfer learning method, we developed a deep learning system based on COVID-19 CT images and non-COVID-19 CT images, and the model had an average accuracy of 76.27% for classifying the two different CT images. Subsequently, we used the adversarial attack algorithm FGSM to show that the COVID-19 CT image-based deep learning system had security vulnerabilities. When FGSM was employed for the attack, the pretrained model’s identification accuracy for non-COVID-19 CT images fell from 80% to 0%. In the field of AI security, there has been a lot of research on the security of deep learning systems based on natural images. Unlike natural-image-based deep learning systems, the security and reliability of medical deep learning systems are critical to every patient; therefore, findings ways of building a safe, reliable, and trustworthy medical imaging system is a very important issue.

**Figure 6:**
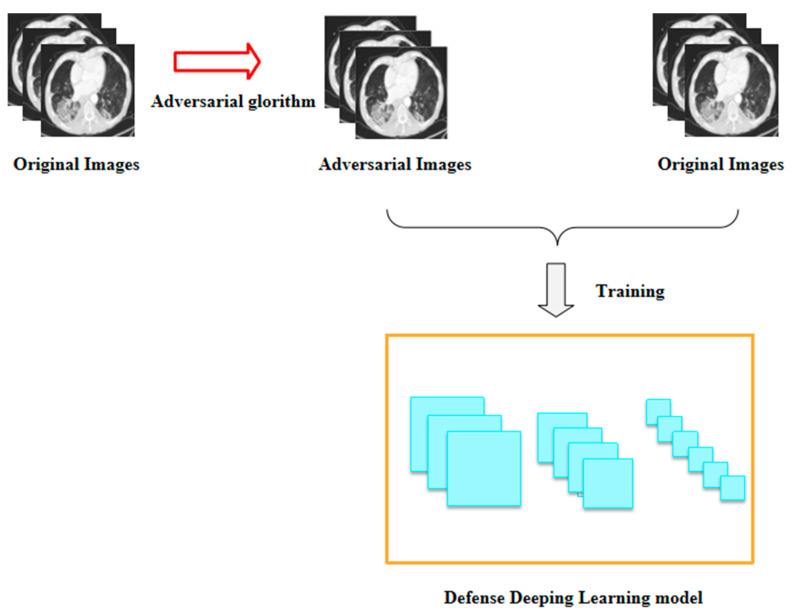
The adversarial training pipeline of the COVID-19 CT image-based deep learning system.

To solve this problem, we used adversarial images to strengthen the model, as shown in Figure 6. First of all, we generated a large number of adversarial images by attacking the target model using the adversarial attack algorithm, and then we put the adversarial images into the model for retraining together with the original data, so that the deep learning model could learn the features of the adversarial images during the training process, and thus could continuously update various parameters in the model to achieve better performance. In this way, we obtained a defense model based on COVID-19 CT images.

**Figure 7:**
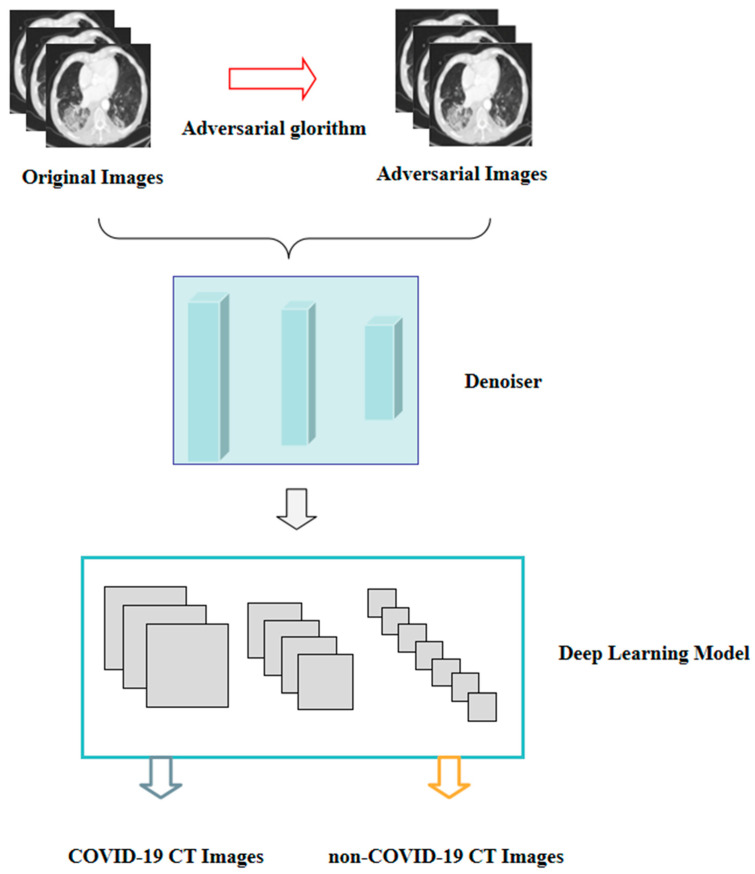
The pipeline of the noise detector COVID-19 CT image-based deep learning system.

In addition, another approach may involve adding a denoiser to the COVID-19 CT image-based deep learning system (Figure 7). An image is made up of useful information and noise that degrades clarity. The function of a denoiser is to remove the noise from the image and to retain only the useful information for a clear image. Deep learning is the process of extracting various features of the input data, and then continuously updating the parameters to achieve the desired performance. Before the training process, a denoiser can be added to preprocess all the images, which can then reduce the noise of the adversarial images, thus, minimizing the interference of the adversarial images to the training model and improving the accuracy of the deep learning model.

## 6. Conclusions

There is no doubt that the application of deep learning in medical diagnosis is promising, and AI technology has contributed greatly to the rapid development of medicine and health care. However, issues such as safety and reliability in deep learning systems cannot be ignored, especially in the medical field where people’s health is crucial. In this paper, we used COVID-19 CT images and non-COVID-19 CT images to address the vulnerabilities and security issues of a deep learning system, and then discussed how a more secure and reliable deep learning system could be built to address the security vulnerabilities. Most importantly, in the real world, one of the security risks of a COVID-19 CT image-based deep learning system is medical fraud created by modifying non-COVID-19 CT images into COVID-19 CT images to obtain high health insurance premiums. We hope to draw developers’ attention to the security and reliability of deep learning systems so that they can develop more secure and reliable medical-based deep learning systems.

However, in this paper, we do not engage in extensive experimental research; we merely discuss two strategies for defense against adversarial attacks. It is more crucial, in our opinion, to learn how to better defend against adversarial attacks and to create a more secure and reliable COVID-19 CT image-based deep learning system. As a result, more thorough studies should be conducted on the defense deep learning system in future work. In other words, attack and defense are similar to a wrestling match against each other, They grow and improve in the constant confrontation, which leads to better robustness of the deep learning model. For medical images, slight interference can cause incredible differences in judgment results, and medical images are closely related to people’s health. Therefore, the security and reliability of deep learning systems based on medical images are particularly important. In addition, various adversarial attacks are developed based on natural images, and although the attack algorithms can also be applied to medical images, there are no adversarial attack algorithms specific to medical images. There is a significant difference between natural images and medical images. Medical images have more special textures and features as compared to natural images, and the different features reflect the degree of illness; thus, developing adversarial attack algorithms based on medical images is also very important.

## Figures and Tables

**Table 1:** The classification of adversarial attack algorithms.

Method	Attack	Attack Box	Attack Target
Optimization-based method	JMSA [47]	White	Target
	L-BFGS [48]	White	Target
	C&W	White	Target
Gradient-based method	FGSM	White	Target
	BIM	White	No target
	PGD	White	No target
	MIM [49]	White	No target
Adversarial network-based method	AdvGAN	White–black	No target
	AdvGAN++ [50] AdvFaces [51]	White–black White–black	No target No target

**Table 2:** The classification of datasets.

Dataset	COVID-19 CT Images	Non-COVID-19 CT Images
Training set	279	317
Validation set	35	40
Testing set	35	40
Total	349	397

**Table 3:** The classification accuracy (%) and AUC (%) of the COVID-19 CT image-based deep learning model.

	Accuracy	AUC
COVID-19 CT image deep learning model	76.27	85.80

**Table 4:** The effect of epsilons on the accuracy of non-COVID-19 image classification.

Epsilons	Predicted Correct	Total CT Images	Accuracy
0	32	40	0.800
0.1	32	40	0.800
0.2	31	40	0.775
0.3	30	40	0.750
0.4	22	40	0.550
0.5	19	40	0.475
0.6	14	40	0.350
0.7	7	40	0.175
0.8	3	40	0.075
0.9	0	40	0.000
1.0	0	40	0.000

## Data Availability

The data presented in this study are available upon request from the corresponding author.
